# Molecular engineering of Cyanine–Hoechst hybrid materials for nanoscale DNA and chromatin imaging

**DOI:** 10.1088/2515-7639/ae857f

**Published:** 2026-07-16

**Authors:** Amit Kumar Singh, Md Abul Shahid, Yeting Zheng, Andrea Tomassini, Xiayi Gong, Shohag Chandra Das, Giulio Palummieri, William M Piedra, Hao F Zhang, Diana Velluto, Françisco M Raymo, Yang Zhang

**Affiliations:** 1 Frost Institute for Chemistry and Molecular Science, University of Miami, 1201 Memorial Drive, Coral Gables, FL 33146, United States of America; 2 Molecular Analytics and Photonics (MAP) Lab, Department of Textile Engineering, Chemistry and Science, North Carolina State University, Raleigh, NC 27606, United States of America; 3 Department of Biomedical Engineering, Northwestern University, 2145 Sheridan Road, Evanston, IL 60208, United States of America; 4 Diabetes Research Institute, Miller School of Medicine, University of Miami, 1450, NW 10th Avenue, Miami, FL 33136, United States of America; 5 Lampe Joint Department of Biomedical Engineering, North Carolina State University and University of North Carolina at Chapel Hill, Raleigh, NC 27695, United States of America

**Keywords:** BODIPY dyes, fluorescence nanoscopy, DNA labeling, chromatin nanostructure, single-molecule spectroscopy

## Abstract

Chromatin is a hierarchically organized soft material whose nanoscale structure and heterogeneity regulate essential genomic functions. Resolving this organization requires molecular imaging materials that combine selective DNA binding with photophysical properties compatible with nanoscale localization and energy transfer under biologically relevant conditions. Conventional bisbenzimide (Hoechst) DNA stains provide robust targeting of nuclear DNA but limited applicability for super-resolution imaging. Here, we investigate the modular molecular engineering strategy in which cyanine chromophores (Cyanine3, Cyanine5, or Cyanine7) are covalently integrated with a bisbenzimide DNA-binding motif through an aliphatic spacer to form hybrid fluorescent materials. This design decouples DNA recognition from optical functionality, allowing independent optimization of binding affinity and photophysical performance. In the resulting conjugates, the cyanine units retain their intrinsic brightness and spectral properties, while the bisbenzimide ligand preserves high-affinity minor-groove binding to nuclear DNA. The Cyanine3–Hoechst and Cyanine5–Hoechst hybrids enable high-contrast imaging of nuclear DNA in fixed and permeabilized cells using long-wavelength excitation (>500 nm), with strongly suppressed extranuclear background. The substantial spectral overlap between Cyanine3 emission and Cyanine5 absorption further enables efficient Förster resonance energy transfer within the nuclear environment, providing a route to probe nanoscale proximity and organization in chromatin. Owing to their favorable photophysical stability and brightness, these hybrid materials also support single-molecule localization microscopy, revealing nanostructured features within the nucleus that are not resolved in diffraction-limited images. This approach provides a versatile platform for developing next-generation imaging materials tailored to the study of chromatin as a dynamic soft matter system.

## 1. Introduction

**Figure 1.**
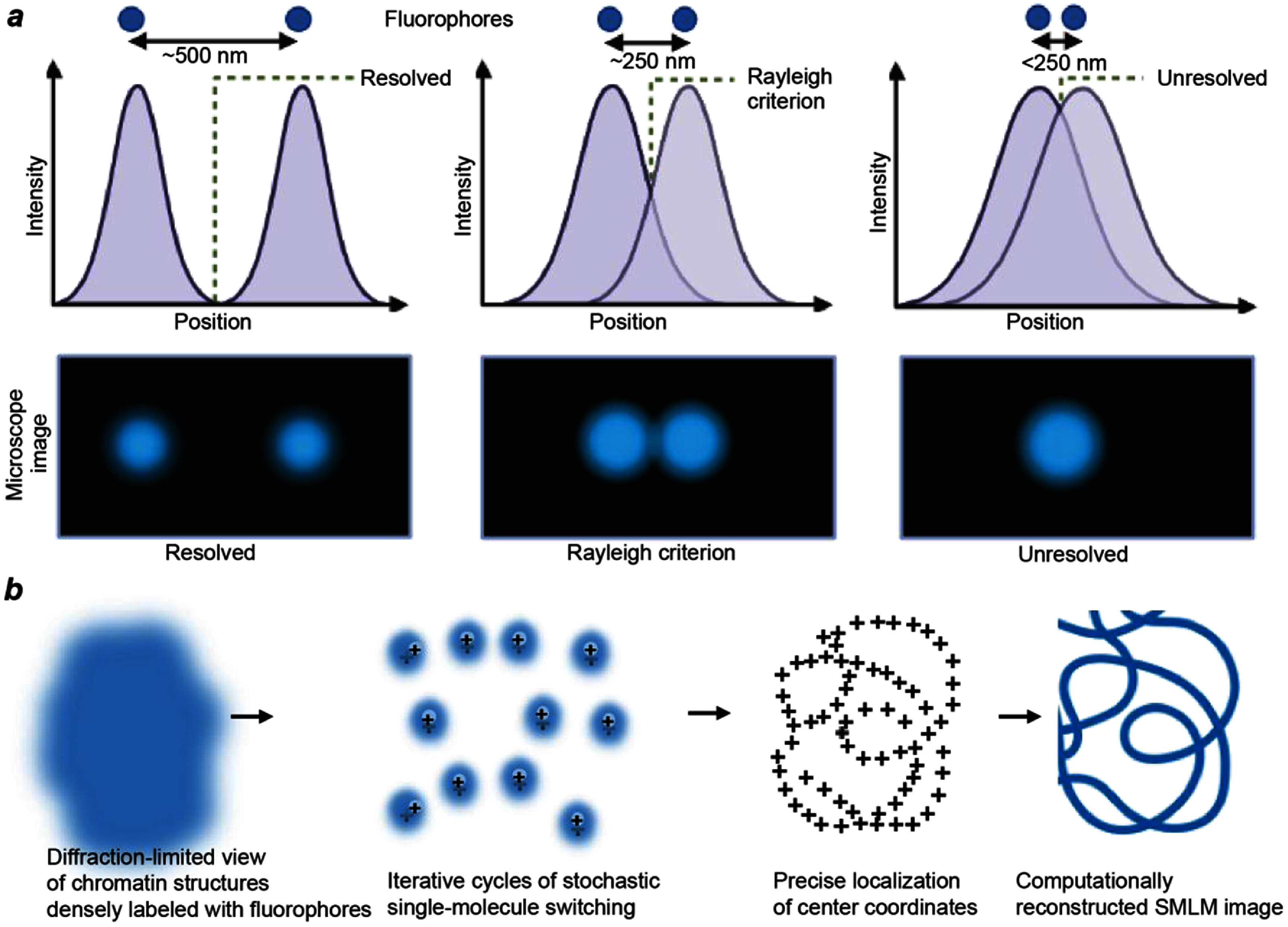
Overview of SMLM principle for nanoscopic imaging of chromatin structure.

The spatial organization of DNA into chromatin represents a hierarchically structured soft material whose nanoscale architecture plays a central role in regulating gene expression, replication, and genome stability [1, 2]. Chromatin organization emerges from multiscale interactions between DNA, histone proteins, and associated factors, giving rise to domains with distinct physical properties, packing densities, and dynamics [3, 4]. Visualizing these features at the molecular scale therefore requires imaging probes that not only bind DNA with high specificity but also possess photophysical characteristics compatible with nanoscale localization under biologically relevant conditions [5]. Single-molecule localization microscopy (SMLM) [6–8] achieves sub-diffraction resolution by temporally separating the emission of individual fluorophores and localizing their centroids with nanometer precision (figure 1(a)). For chromatin imaging, this approach enables direct visualization of chromatin fibers [3], domain boundaries, and nanoscale organization that are otherwise obscured in ensemble averaged measurements (figure 1(b)). Crucially, the performance of SMLM is dictated not only by the optical instrumentation but also by the molecular design of the fluorescent probes themselves, including brightness, photostability, switching behavior, and compatibility with the local chemical environment [5, 9, 10]. From a materials perspective, chromatin imaging by SMLM thus places stringent requirements on fluorophores as functional molecular materials operating within a crowded, heterogeneous, and dynamic polymeric matrix [11–14].

Conventional strategies to meet these requirements have focused on the direct design of fluorescent DNA binders in which the chromophore itself is engineered to recognize DNA while simultaneously providing favorable photophysical properties. Specifically, Hoechst dyes constitute a well-established class of small molecule DNA binders built around a bisbenzimide scaffold bearing a methylated piperazinyl substituent at one terminus and a substituted phenyl ring at the other [15–17]. This molecular architecture promotes noncovalent binding to the DNA minor groove, with a strong preference for A–T rich sequences, accompanied by a pronounced fluorescence enhancement upon binding. The increase in emission arises primarily from changes in chromophore solvation and conformational restriction within the DNA groove, enabling high signal to noise nuclear staining [11, 18, 19]. Owing to these properties, Hoechst dyes together with 4’,6-Diamidino-2-phenylindole (DAPI) [20] have remained standard reagents for nuclear DNA labeling for more than five decades [15–17, 21, 22].

Despite their widespread use, the photophysical characteristics of Hoechst dyes impose fundamental limitations for nanoscale fluorescence imaging. Their excitation in the ultraviolet or violet spectral region (<410 nm) [16, 20] leads to increased cell damage [23], photobleaching, and background autofluorescence. These constraints are particularly problematic for chromatin nanoscale imaging, where resolving nanoscale structural heterogeneity [24] and dynamic rearrangements demands prolonged acquisition and precise localization of individual emitters.

**Figure 2.**
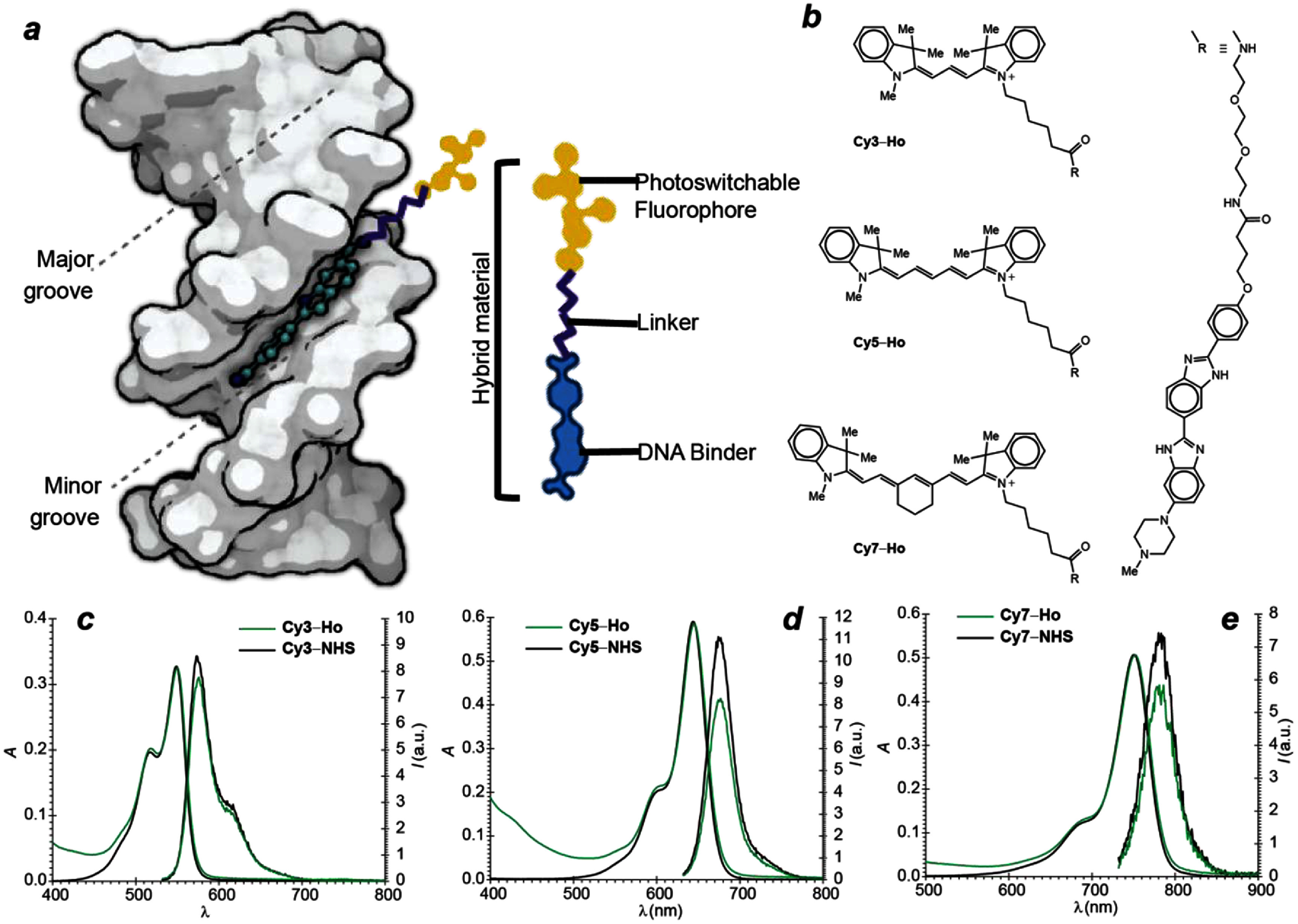
The modular design of a hybrid DNA-binding photoswitchable probe system and its binding to the minor groove of DNA (a). Molecular structures (b) together with absorption and emission spectra (c)–(e) of the Cyanine–Hoechst conjugates (**Cy3–Ho, Cy5–Ho**, and **Cy7–Ho**).

An alternative and less explored paradigm is to treat DNA recognition and fluorescence emission as modular functions (figure 2(a)), combining them into hybrid molecular materials through covalent assembly [16]. In this hybrid strategy, Hoechst serves as a robust and well characterized DNA targeting module, while a second chromophore provides the desired optical functionality. Realizing this concept requires careful molecular engineering of the linker connecting the two components, such that DNA binding by the bisbenzimide motif is preserved and electronic or conformational perturbations of the emissive unit are minimized. Previous studies have demonstrated the feasibility of this approach using BODIPY [25–29], cyanine [30, 31], and xanthene fluorophores [25, 31–40], enabling super resolution imaging of nuclear DNA by SMLM [31, 33, 34, 38, 39]and stimulated emission depletion microscopy [32, 36, 40]. These examples highlight the promise of hybrid molecular probes as tunable materials for nanoscale bioimaging.

Here, we extend the modular hybrid-material concept by engineering a family of Cyanine–Hoechst conjugates in which the length of the polymethine bridge is systematically varied to tune spectral properties across the visible and near-infrared regions. We report the synthesis, photophysical characterization, and cellular imaging performance of these hybrid materials and evaluate their utility for nanoscale imaging of nuclear DNA and chromatin architecture. While fluorophore–Hoechst conjugates have previously been demonstrated, a systematic materials-level investigation of structure–property relationships within such hybrids remains lacking. In particular, the influence of fluorophore identity and polymethine length on DNA-binding specificity has not been rigorously examined, nor has the reciprocal effect of the DNA-binding module on the photophysical and photochemical behavior of the cyanine chromophore been quantitatively assessed. These coupled interactions are critical for determining brightness, stability, spectral fidelity, and imaging performance within the complex nuclear environment. This work directly addresses this gap by elucidating how chromophore structure and DNA-binding functionality mutually influence one another, establishing design principles that govern the performance of hybrid fluorescent materials for chromatin imaging.

## 2. Results and discussion

### 2.1. Design and synthesis

A synthetic strategy was designed to connect the NHS esters of Cyanine3, Cyanine5, and Cyanine7 fluorophores (**Cy3–NHS, Cy5–NHS**, and **Cy7–NHS**) to a bisbenzimide ligand (**9**), bearing an aliphatic tail on its phenyl ring with a terminal primary amino group (figures 2(b) and S1). The tetrafluoroborate salts of the three NHS esters are available from commercial sources. The bisbenzimide precursor can be prepared in nine steps from commercial starting materials, adapting literature procedures [31]. Reaction of stoichiometric amounts of **9** with each NHS ester in anhydrous dimethylsulfoxide (DMSO), under the assistance of di-*i*-propylethylamine (DIPEA), gave the corresponding Cyanine–Hoechst conjugate (**Cy3–Ho, Cy5–Ho**, and **Cy7–Ho** in figure 2) in a yield of 50% after preparative thin-layer chromatography. Electrospray ionization mass spectrometry of the isolated products revealed peaks at *m/z* of 1079.6323, 1105.6390, and 1171.6861 Da respectively for the corresponding molecular ion ([M]+). Consistently, ^1^H nuclear magnetic resonance spectroscopy showed the characteristic peaks for the hydrogen atoms of the cyanine and bisbenzimide components in each instance, confirming that the two chromophoric systems are integrated in the same molecular construct.

### 2.2. Photophysical characterization

The ensemble absorption and emission spectra of **Cy3–Ho, Cy5–Ho**, and **Cy7–Ho** (green traces in figures 2(c)–(e)) in aerated ethanol show the characteristic bands of the corresponding cyanine chromophore with a noticeable, and expected, bathochromic shift with the increase in polymethine length. Comparison to the spectra of **Cy3–NHS, Cy5–NHS**, and **Cy7–NHS** (black traces in figures 2(c)–(e)) reveals negligible differences in the spectral profile of both bands in all instances. Minor decreases in fluorescence quantum yield (*φ*_F_ in table 1) occurs with Hoechst conjugation for all cyanine chromophores. These observations suggest that the covalent attachment of the bisbenzimide ligand to each one of the three cyanine chromophores has minimal influence on their photophysical properties.

**Table 1.** Photophysical parameters of designed DNA-targeting fluorescent materials^a^.

*Compound*	$\lambda $*_Ab_* (nm)	$\lambda $*_Em_* (nm)	$\Delta \lambda $(nm)	φ_F_
Cy3–NHS	549	574	25	0.31
Cy3–Ho	549	576	27	0.28
Cy5–NHS	644	674	30	0.20
Cy5–Ho	645	674	29	0.15
Cy7–NHS	751	785	34	0.30
Cy7–Ho	751	785	35	0.23

^a^ The wavelengths at the absorption (*λ*_Ab_) and emission (*λ*_Em_) maxima, Stokes’ shift (Δ*λ*), and fluorescence quantum yield (*φ*_F_) of each compound were measured in aerated EtOH at 25 °C. The values of *φ*_F_ listed for the tetrafluoroborate salts of the NHS esters are from the manufacturer (Lumiprobe Corporation, lumiprobe.com). The values of *φ*_F_ reported for the Hoechst conjugates were measured relative to those of the NHS esters.

### 2.3. Confocal laser scanning microscopy (CLSM)

**Figure 3.**
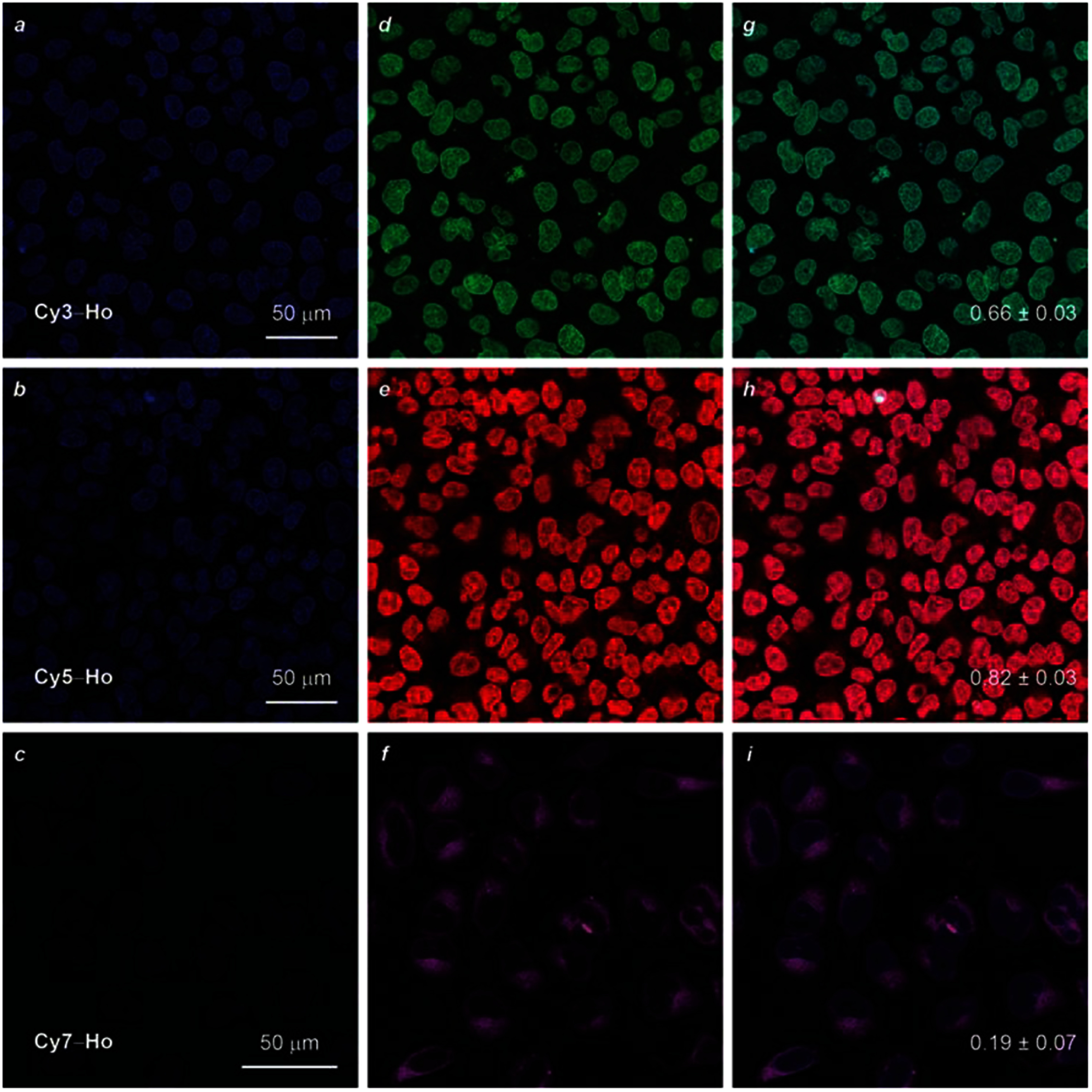
CLSM images (a)–(c): *Ex.* = 405 nm, *Em.* = 425–479 nm; (d): *Ex.* = 514 nm, *Em.* = 523–600 nm; (e): *Ex.* = 600 nm, *Em.* = 661–739 nm; (f): *Ex.* = 745 nm, *Em.* = 759–834 nm of fixed and permeabilized U2OS cells stained with **Cy3–Ho** (a), (d), and (g), **Cy5–Ho** (b), (e), and (h), or **Cy7–Ho** (c), (f), and (i) and the corresponding overlays (g)–(i) with PCC values for the fluorescence co-localization in the two overlayed channels.

CLSM images of fixed and permeabilized U2OS cells, incubated with **Cy3–Ho, Cy5–Ho**, or **Cy7–Ho**, show exclusively nuclear fluorescence (figures 3(a)–(c)) upon excitation of the Hoechst ligand of each compound at 405 nm and detection in the violet–blue region of the electromagnetic spectrum. Indeed, only after association with DNA can the Hoechst chromophore produce significant fluorescence. The corresponding images captured with excitation of the Cyanine3, Cyanine5, or Cyanine7 chromophore instead and detection in the green–orange, red, or near-infrared region respectively, reveal fluorescence almost exclusively inside the nucleus for **Cy3–Ho** and **Cy5–Ho** (figures 3(d) and (e)) but predominantly outside for **Cy7–Ho** (figure 3(f)). Overlays of the Hoechst and cyanine channels further confirm fluorescence co-localization in the nucleus for **Cy3–Ho** and **Cy5–Ho** (figures 3(g) and (h)) with Pearson’s correlation coefficient (PCC) values of 0.66 ± 0.03 and 0.82 ± 0.03 respectively. By contrast, the emission of Cyanine7 appears mostly in the cytosol (figure 3(i)), where the Hoechst ligand remains essentially not emissive. As a result, the PCC value for **Cy7–Ho** is only 0.19 ± 0.03. Presumably, the relatively long polymethine bridge of Cyanine7 promotes the non-specific adsorption of **Cy7–Ho** on lipophilic intracellular compartments, limiting the ability of this large and hydrophobic molecular construct to label the cellular nucleus selectively. Instead, **Cy3–Ho** and **Cy5–Ho** penetrate the nuclear envelope effectively to highlight nuclear DNA selectively with negligible background fluorescence from the cytoplasm.

**Figure 4.**
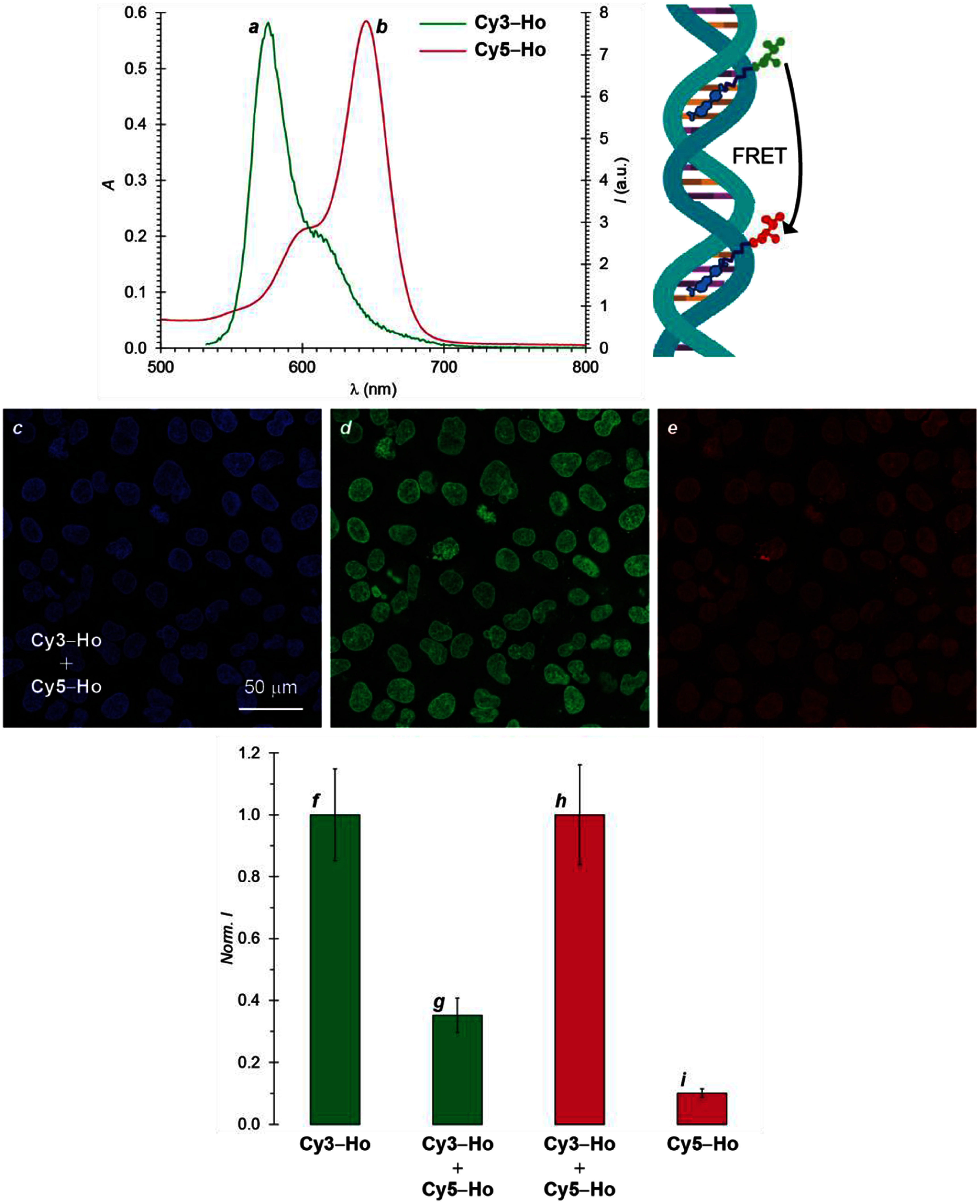
Absorption spectrum (a) of Cy3–Ho (4 *µ*M) and emission spectrum (b) of Cy5–Ho (1 *µ*M, *Ex*. = 620 nm) in aerated EtOH at 25 °C along with a schematic illustration of the FRET process between Cy3–Ho and Cy5–Ho upon both binding to DNA. CLSM images (c): *Ex*. = 405 nm, *Em*. = 425–479 nm; (d): *Ex*. = 514 nm, *Em*. = 523–600 nm; (e): *Ex*. = 514 nm, *Em*. = 661–739 nm) of fixed and permeabilized U2OS cells co-stained with equimolar amounts of Cy3–Ho and Cy5–Ho. Average emission intensities with standard deviations measured for the Cyanine3 (green bars, *Ex*. = 514 nm, *Em*. = 523–600 nm) and Cyanine5 (red bars, *Ex*. = 514 nm, *Em*. = 661–739 nm) chromophores in CLSM images of cells incubated with only one of the two dyes (f) and (i) or equimolar amounts of both (g) and (h).

Comparison of the emission spectrum of **Cy3–Ho** to the absorption spectrum of **Cy5–Ho** shows the bands (figures 4(a) and (b)) of the two cyanine chromophores to overlap significantly. The corresponding overlap integral and Förster distance are 1.03 × 10^12^ M^–1^ nm^3^ and 6.1 nm respectively. These observations suggest that Förster resonance energy transfer (FRET) from **Cy3–Ho** to **Cy5–Ho** should occur within the nuclear envelope, if the two dyes co-localize in close proximity. To assess these effects, fixed and permeabilized U2OS cells were incubated with equimolar amounts of **Cy3–Ho** and **Cy5–Ho**, under otherwise identical conditions of the experiments performed with the individual dyes. CLSM images (figures 4(c)–(e)) of the co-incubated cells reveal nuclear colocalization of the fluorescence for the Hoechst ligand and the two cyanine chromophores. Their comparison to images of cells stained with the individual dyes, however, shows the emission intensity of the Cyanine3 chromophore (figures 4(f) and (g)) to decrease and that of the Cyanine5 chromophore (figures 4(h) and.(i)) to increase with co-incubation. The fluorescence of the Hoechst chromophore was used to normalize the emission intensities of donor and acceptor in the FRET measurement. These fluorescence changes are consistent with energy transfer from **Cy3–Ho** to **Cy5–Ho**, suggesting that their stochastic distribution in the nuclear envelop results in proximal co-localization of the two dyes. Specifically, the decrease in donor emission (figures 4(f) and (g)) corresponds to a FRET efficiency of ∼65% and an average donor—acceptor distance of ∼5.5 nm.

### 2.4. Single-molecule localization microscopy

**Figure 5.**
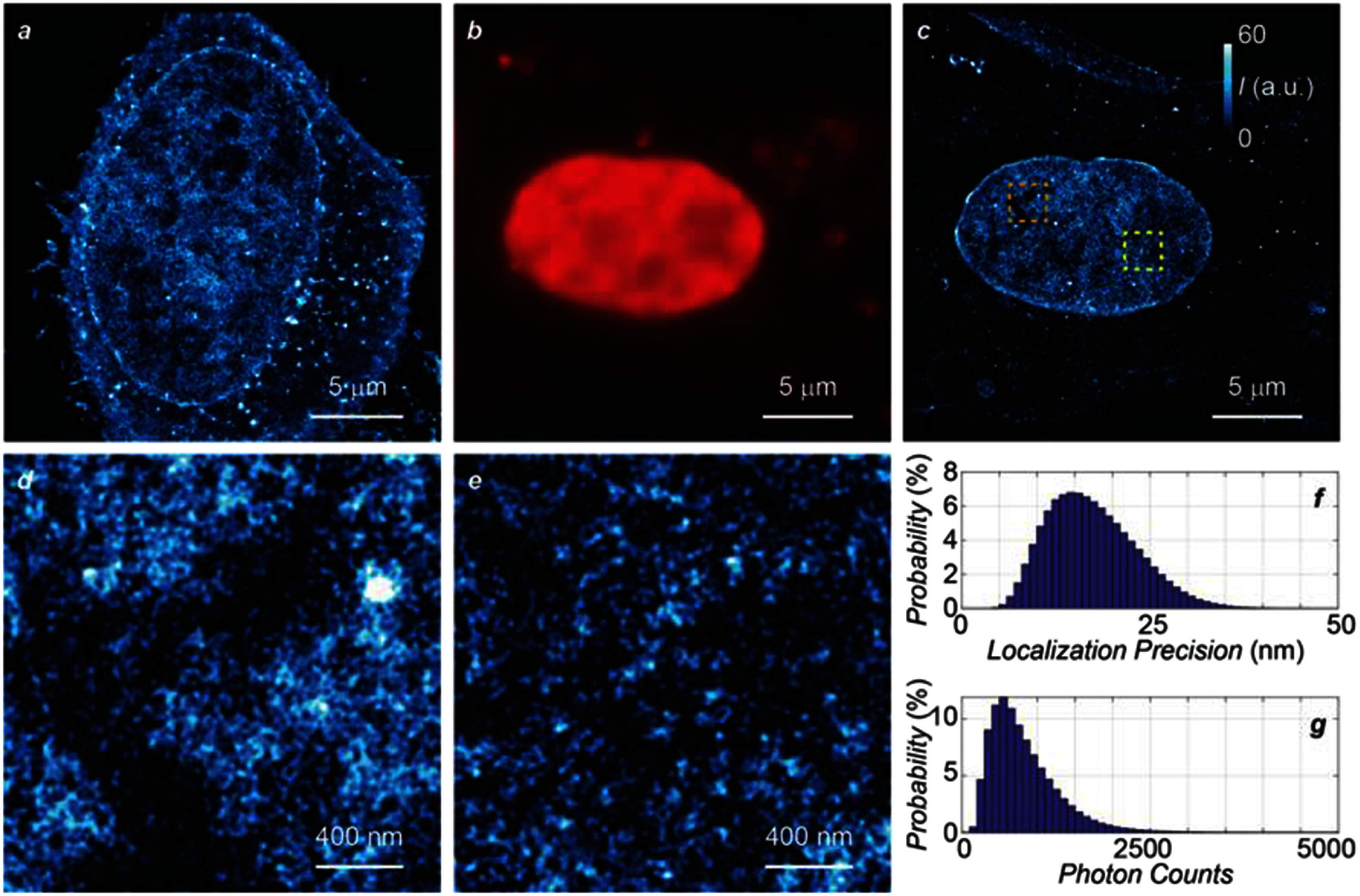
SMLM (a) and (c)–(e) and epifluorescence (b) images of fixed and permeabilized U2OS cells stained with **Cy5–Ho**. (a) and c are images of cells stained without and with the extraction step respectively. (b) and (c) are images of the same cell. (d) and (e) are magnified views of the orange and yellow boxes in c. Probability distributions of the localization precision (f) and photon counts (g) measured for 1048 575 single molecules in c.

The high labeling density greatly enhances the quality of SMLM imaging. SMLM was initially performed on U2OS cells fixed, permeabilized, and stained with **Cy5–Ho** using a protocol similar to that of the CLSM experiments. In the resulting SMLM image (figure 5(a)), localized fluorescence can be visualized throughout the intracellular space and within the nuclear envelope in contrast to the CLSM image (figure 3(e)), which revealed almost exclusively nuclear fluorescence. The discrepancy between the two imaging modalities, under the same labeling protocol, is likely a result of the ultrahigh single-molecule sensitivity of the electron-multiplying charge-coupled device camera in the SMLM system. With such a high sensitivity, minor non-specific labeling can be detected and subsequently visualized in SMLM, while it remains undetected in CLSM. Indeed, this effect is known and, to overcome it, the labeling protocol must be optimized specifically for SMLM [41].

To minimize non-specific extranuclear adsorption, an extraction step with Triton X-100 (0.4% v/v) in PBS was performed before cell fixation, following a standard literature procedure. This additional step is expected to remove most of the hydrophobic components from the cytosol, before incubation of the cells with the dyes, minimizing the non-specific adsorption of the fluorescent probes. Changes in dye concentration (0.1–10 *µ*g ml^−1^) and incubation time (5–60 min) were also explored to find that treating the cells with 1 *µ*g ml^−1^ of the fluorescent probe for 15 min only ensures optimal nuclear labeling with negligible extranuclear adsorption. The diffraction-limited epifluorescence image (figure 5(b)) of the same cell shows the overall nuclear shape with unresolved structural features in its interior. In contrast, the corresponding SMLM image (figure 5(c)) enables the unambiguous visualization of the cellular nucleus with only scattered signals outside. Magnified views (figures 5(d) and (e)) of this image reveal two distinct sub-nuclear regions. Within each sub-nuclear region, different nanoscale patterns and clusters with sizes varying from tens to hundreds of nanometers can be observed. The signals are partitioned in two bright regions, separated by a dark area, in one magnification (figure 5(d)) and uniformly distributed throughout the field of view in the other (figure 5(e)). These distinct nanoscale structural variations presumably imply high-order chromatin structures, in agreement with a literature precedent [31]. The corresponding probability distributions of the single-molecule localization precision (figure 5(f)) and photon count (figure 5(g)) give average values of ∼17 nm and ∼1075 respectively. These results demonstrate that the labeling capabilities and photophysical properties of **Cy5–Ho**, in conjunction with the optimized protocol for nuclear staining, allow the direct visualization of chromatin nanostructures at a spatial resolution that is not accessible with conventional diffraction-limited fluorescence microscopy.

SMLM images were recorded also with **Cy3–Ho** and **Cy7–Ho**, under the same conditions of those captured with **Cy5–Ho**, for comparison. Once again, the optimized staining protocol ensured minimal non-specific adsorption in the cytosol for **Cy3–Ho** (figures S2a and S2b) and **Cy7–Ho** (figures S3a and S3b). In both instances, the epifluorescence image (figures S2a and S3a) shows the characteristic cyanine fluorescence within the nuclear envelope and a dark area also in the nucleus, which most likely is the nucleolus. The corresponding SMLM images (figures S2b, S2c, S3b and S3c), however, differ from those acquired with **Cy5–Ho** (figures 5(c)–(e)). Large (>200 nm) and sparsely-distributed fluorescent clusters are clearly evident in the case of Cy3–Ho and Cy7–Ho. The contrasting behavior of Cy5–Ho is likely attributable to differences in single-molecule photophysics, particularly blinking behavior, rather than differences in nuclear labeling efficiency [42]. More specifically, Cy3–Ho requires pre-bleaching before the SMLM acquisition because of the large numbers of molecules in the ‘on’ state which prevents sparse single-molecule localization [42]. The low survival fraction of Cy3 chromophore then resulted in the sparse and insufficient localization patterns across the whole image. On the other hand, Cy7–Ho has more repetitive blinking/localizations before transitioning back to dark states which generate spurious localization clusters that do not represent the underlying molecular distribution [43]. Furthermore, the probability distributions of the localization precisions for **Cy3–Ho** (figure S2d) and **Cy7–Ho** (figure S3d) show average values of only ∼26 and ∼30 nm respectively, even though their photon counts are similar to and greater than that for **Cy5–Ho** respectively (figure 5(g)**, S2e and S3e**). Thus, these observations demonstrate that **Cy5–Ho** significantly outperforms **Cy3–Ho** and **Cy7–Ho** in the SMLM imaging of nuclear DNA.

### 2.5. Single-molecule spectroscopy and heterogeneity

The evident intranuclear labeling of **Cy3–Ho** and **Cy5–Ho** (figure 4) and their efficient FRET indicate that both dyes bind nuclear DNA with proximal localization. Such binding events are expected to alter the environment around the respective cyanine chromophores and affect their spectral behavior. To probe these effects, single-molecule spectroscopy of **Cy3–Ho** and **Cy5–Ho** was performed within nuclear regions, using our previously reported spectroscopic SMLM setup [44].

**Figure 6.**
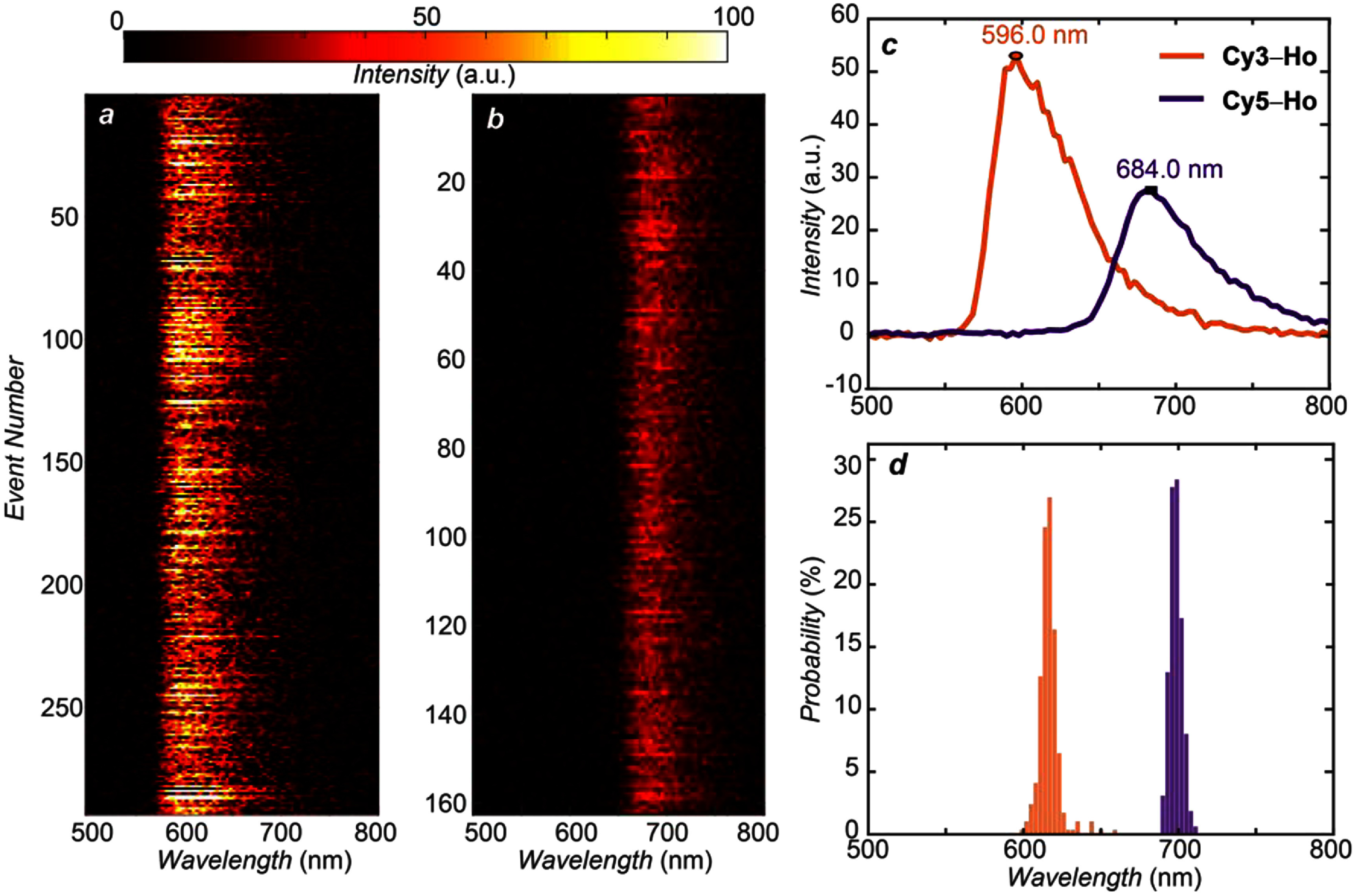
Representative single-molecule fluorescence spectra of 294 **Cy3–Ho** molecules (a) and 163 **Cy5–Ho** molecules (b) inside the nucleus of fixed and permeabilized U2OS cells with the corresponding averaged single-molecule spectra (c) and probability distributions (d) of the single-molecule spectral centroids for **Cy3–Ho** (orange trace/bars) and **Cy5–Ho** (purple trace/bars).

Under similar imaging conditions to SMLM, hundreds of single-molecule spectra (figures 6(a) and (b)) of **Cy3–Ho** and **Cy5–Ho** were captured in minutes and pronounced spectral variations/shifts were observed for each of the two dyes. Their averaged single-molecule spectra (figure 6(c)) resemble the ensemble profiles (figure 2) with emission peaks at 596 and 684 nm and bathochromic shifts of 20 and 10 nm, relative to the ensemble emission peaks (table 1), respectively. The 10 nm shift observed for **Cy5–Ho** is expected when the environment around the Cyanine5 chromophore changes from an organic solvent to a biological/aqueous medium [45]. In contrast, the 20 nm shift detected for **Cy3–Ho** cannot simply be explained by solvent effects and might suggest the co-existence of different interaction modes between this dye and DNA. The probability distributions of the intensity-weighted spectral centroid among all measured molecules (figure 6(d)) have mean values of 617 and 699 nm with standard deviation (STD) of 6.6 and 3.8 nm for **Cy3–Ho** and **Cy5–Ho** respectively. The differences in the STD values may stem from the dyes’ distinct intrinsic single-molecule spectral heterogeneity, variations in their local microenvironments within the nuclear envelope, or a combination of both factors. These influences can alter intra- and intermolecular coordinates, thereby enhancing spectral variability at the single-molecule level [46]. However, the high FRET efficiency indicates that the two dyes reside within the same nuclear region. Consequently, the observed STD differences primarily reflect intrinsic single-molecule fluorescence spectral heterogeneity (smFLUSH) of the fluorophores rather than environmental artifacts. Notably, the STD value measured for **Cy5–Ho** is consistent with previously reported values [47] for other far-red cyanine dyes across different environments, further supporting its intrinsic smFLUSH characteristics. In summary, single-molecule spectroscopy in the cell nucleus reveals distinct smFLUSH signatures for **Cy3–Ho** and **Cy5–Ho**, potentially indicating different DNA-binding modes for the two dyes.

## 3. Conclusion

We have demonstrated that straightforward amide coupling enables the modular integration of cyanine fluorophores with a bisbenzimide DNA-binding motif to yield a family of Cyanine–Hoechst hybrid materials with systematically varied polymethine bridge lengths. Despite covalent fusion of the two functional components, the cyanine chromophores largely retain their intrinsic absorption and emission characteristics, exhibiting only modest reductions in quantum yield, while the Hoechst unit preserves its high-affinity association with nuclear DNA.CLSM confirms efficient nuclear targeting for the Cyanine3 and Cyanine5 conjugates under long-wavelength excitation, whereas the more hydrophobic Cyanine7 derivative preferentially accumulates in the cytosol, highlighting the influence of chromophore structure on intracellular transport and nuclear accessibility. Co-staining experiments further reveal efficient FRET (∼65%) between Cyanine3–Hoechst and Cyanine5–Hoechst in the nucleus, corresponding to an average donor–acceptor separation of ∼5.5 nm. Future studies incorporating quantitative FRET analyses, including fluorescence lifetime measurements and controlled donor-to-acceptor stoichiometries, will be valuable for rigorously characterizing the energy-transfer process and refining distance estimates in cells.

SMLM confirms nuclear localization of all three constructs and reveals nanostructured features of the nuclear envelope not resolved under diffraction-limited imaging. Among the series, the Cyanine5–Hoechst conjugate exhibits superior localization precision (∼17 nm) and more uniform nanoscale distributions, whereas the Cyanine3 and Cyanine7 analogues show larger apparent clustering, likely reflecting differences in photophysical duty cycles. Spectroscopic SMLM further uncovers distinct environmental heterogeneity experienced by Cyanine3 relative to Cyanine5 within the nuclear interior, demonstrating the sensitivity of hybrid probes to chromatin microenvironments.

In summary, we have developed three Cyanine–Hoechst conjugates as color-tunable hybrid materials for SMLM imaging of nuclear DNA in fixed cells. This work validates a versatile and general molecular engineering strategy that repurposes Hoechst as a targeting ligand for photoswitchable fluorophores, paving the way for advanced nanoscopic imaging of chromatin architecture.

## Data Availability

All data that support the findings of this study are included within the article (and any supplementary files). Supplementary Information available at https://doi.org/10.1088/2515-7639/ae857f/data1.
